# Acute Effects of Transcranial Direct Current Stimulation Combined with High-Load Resistance Exercises on Repetitive Vertical Jump Performance and EEG Characteristics in Healthy Men

**DOI:** 10.3390/life14091106

**Published:** 2024-09-03

**Authors:** Yuping Zhou, Haiting Zhai, Hongwen Wei

**Affiliations:** 1 School of Strength and Conditioning Training, Beijing Sport University, Beijing 100084, China; xihuyuping@163.com; 2 Department of Public Education, Zhejiang College of Construction, Hangzhou 311231, China; 3 School of Basic Sciences for Aviation, Naval Aviation University, Yantai 264001, China; minghuhaiting@163.com; 4 School of Sports Coaching, Beijing Sport University, Beijing 100084, China; 5 Key Laboratory for Performance Training & Recovery of General Administration of Sport, Beijing Sport University, Beijing 100084, China

**Keywords:** transcranial direct current stimulation, resistance exercises, repetitive vertical jump, electroencephalography

## Abstract

Background: Transcranial direct current stimulation (tDCS) is a non-invasive technique known to enhance athletic performance metrics such as vertical jump and lower limb strength. However, it remains unclear whether combining tDCS with the post-activation effects of high-load resistance training can further improve lower limb performance. Objective: This study investigated the synergistic effects of tDCS and high-load resistance training, using electroencephalography to explore changes in the motor cortex and vertical jump dynamics. Methods: Four experiments were conducted involving 29 participants. Each experiment included tDCS, high-load resistance training, tDCS combined with high-load resistance training, and a control condition. During the tDCS session, participants received 20 min of central stimulation using a Halo Sport 2 headset, while the high-load resistance training session comprised five repetitions of a 90% one-repetition maximum weighted half squat. No intervention was administered in the control group. Electroencephalography tests were conducted before and after each intervention, along with the vertical jump test. Results: The combination of tDCS and high-load resistance training significantly increased jump height (*p* < 0.05) compared to tDCS or high-load resistance training alone. As for electroencephalography power, tDCS combined with high-load resistance training significantly impacted the percentage of α-wave power in the frontal lobe area (F3) of the left hemisphere (F = 6.33, *p* < 0.05). In the temporal lobe area (T3) of the left hemisphere, tDCS combined with high-load resistance training showed a significant interaction effect (F = 6.33, *p* < 0.05). For β-wave power, tDCS showed a significant main effect in the frontal pole area (Fp1) of the left hemisphere (F = 17.65, *p* < 0.01). In the frontal lobe area (F3) of the left hemisphere, tDCS combined with high-load resistance training showed a significant interaction effect (F = 7.53, *p* < 0.05). The tDCS combined with high-load resistance training intervention also resulted in higher β-wave power in the parietal lobe area (P4) and the temporal lobe area (T4) (*p* < 0.05). Conclusions: The findings suggest that combining transcranial direct current stimulation (tDCS) and high-load resistance training significantly enhances vertical jump performance compared to either intervention alone. This improvement is associated with changes in the α-wave and β-wave power in specific brain regions, such as the frontal and temporal lobes. Further research is needed to explore the mechanisms and long-term effects of this combined intervention.

## 1. Introduction

Athletic performance enhancement is driven by diverse factors and a range of training methods designed to foster improvement. However, when advancements stall, some individuals resort to illicit substances to boost brain excitability and overall performance [1]. Consequently, numerous sports scientists are now concentrating on how the central nervous system, particularly the brain, influences athletic performance [2,3]. Given the continuous push to extend physical boundaries in modern competitive sports, emphasizing the importance of training both the body and the brain was crucial. [4]. Exploring the link between neuromodulation techniques and athletic performance, understanding the physiological mechanisms behind performance enhancement, and effectively applying these insights in sports training represented future trends [5].

Transcranial direct current stimulation (tDCS) is a non-invasive technique for brain neuromodulation. It involves applying weak, constant, low-intensity direct currents (1–2 mA) to the scalp to modulate the activity of cortical neurons [6]. tDCS is distinguished by its non-invasive nature, efficiency, user-friendliness, and portability. tDCS has found widespread applications in neurorehabilitation [7,8], the treatment of psychiatric disorders [9,10], skill learning enhancement [11], and memory improvement [12]. Recent studies have also shown that tDCS can effectively enhance athletic performance, improving metrics such as vertical jump, lower limb strength, explosive power, and speed [13,14,15]. Specifically, the vertical jump is the most commonly used measure in research. tDCS has been shown to positively influence vertical jump performance, enhancing peak power, reducing ground contact time, and increasing jump height [16,17,18].

Recently, researchers have introduced the portable Halo Sport 2 headphones, which utilize the principles of transcranial direct current stimulation (tDCS). These headphones contain electrodes that deliver electrical currents to the wearer’s brain, stimulating the motor cortex to enhance neural pathways and increase the firing rate of neural networks [19]. A systematic review and meta-analysis revealed that by combining transcranial direct current stimulation (tDCS) with resistance training, researchers have further enhanced strength capabilities [20]. However, research into the combined effects of tDCS and strength training remains inconclusive. Some studies suggest that combining tDCS with resistance training can significantly enhance muscle strength and lower limb explosive power [21,22,23,24], while others report no significant benefits compared to a sham stimulation combined with resistance training [25,26,27]. These discrepancies could stem from differences in tDCS parameters such as the current intensity, targeted brain areas, duration of stimulation, and electrode size [28]. Additionally, research has demonstrated significant increases in lower limb power output after acute high-load resistance training (≥80% one-repetition maximum, 1RM), marked by shorter ground contact times, higher jumps, and extended airborne phases during vertical jumps [2]. The synergistic potential of high-load resistance training combined with tDCS to enhance lower limb power has not yet been explored.

If combining high-load resistance training with tDCS can significantly enhance lower limb power output, what are the potential mechanisms behind this effect? There are currently no studies reporting on this. Therefore, this study hypothesizes that the combination of resistance training and tDCS can significantly improve repeated jump performance, and that this effect is related to changes in electroencephalography (EEG) characteristics.

## 2. Methods

### 2.1. Participants

Participants were selected based on the following inclusion criteria: (1) Experience in resistance training, with lower extremity strength training at least 3 times/week; (2) male; (3) aged 19–23; and (4) proficiency in large-load half squats and continuous vertical jumps. Additionally, participants were required to have no history of lower extremity or brain injuries or psychiatric illnesses within the last 3 months. Exclusion criteria included individuals with: (1) skin diseases or allergies, (2) fractures or joint injuries in the past six months, (3) no strength training experience, and (4) psychiatric conditions or brain injuries. Thirty-four volunteers initially participated in this study, but only 29 met the experimental requirements and were randomly assigned to one of four experimental conditions: control condition (CON), high-load resistance training (HRT) condition, tDCS intervention (tDCS) condition, and the combination of tDCS and high-load resistance training (tDCS + HRT) condition. Each participant experienced all four conditions in a randomized order. Participants information is shown in Table 1.

### 2.2. Human Ethics and Consent to Participate Declarations

This study strictly adhered to the principles of the Declaration of Helsinki and was approved by the Institutional Review Board (IRB) of Beijing Sport University Ethics Committee (approval number: 2020120H). The review ensured that the research design complied with international and national ethical standards. Prior to the commencement of the experiment, the research team conducted detailed information sessions to ensure all participants fully understood the purpose, expected benefits, potential risks, and their rights within the study. Each participant signed a written informed consent form, which detailed the research procedures, the handling of personal data, and privacy protection measures. To respect participants’ rights, this study ensured that all participants were aware they had the right to withdraw their participation at any stage without providing a reason, and that their decision to withdraw would not affect any services or treatments they were receiving or might receive in the future. The research team committed to immediately removing any information related to a participant’s withdrawal from the study records, and such data would not be used in the analysis.

This study adheres to CONSORT guidelines for reporting clinical trials.

### 2.3. Experimental Design

**Figure 1:**
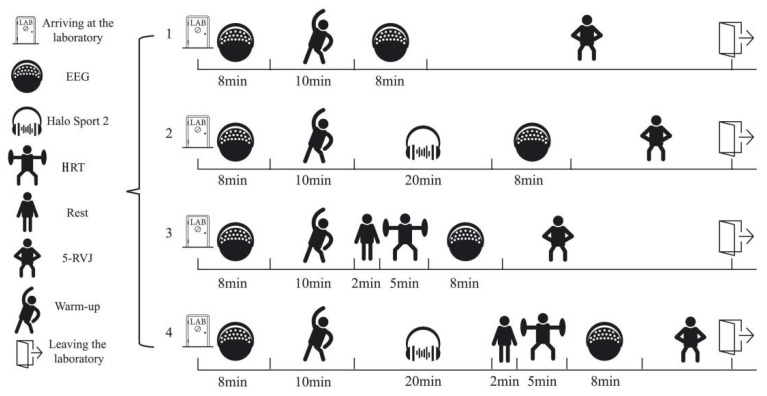
Flow chart of the experiment.

This study employed a single-blind, randomized, crossover design with repeated measures. Each of the 29 participants was randomly assigned to complete four different experimental conditions (CON, HRT, tDCS, and tDCS + HRT) in separate study visits. The order of conditions was counterbalanced to control for potential order effects. The experiments were conducted at the Sports Science Research Centre of Beijing Sport University, China, with each participant visiting the laboratory five times. On the first day, participants had their weight and height measured after emptying their bladders. Subsequently, according to the NSCA testing procedure [29], participants were assessed for half-squat 1RM, performed repeated vertical jumps, and underwent baseline testing. Participants were randomly allocated to complete four tests in a counterbalanced order, with each set scheduled 5 days apart and conducted at the same time of day to control for circadian rhythm influences. Each participant followed a warm-up regimen in every test, consisting of 4 min of jogging, 4 min of dynamic stretching, a 20 m sprint, practice of the vertical jump technique, and 2 min of slow walking. All groups followed a predetermined experimental protocol, as illustrated in Figure 1. Participants in the CON group underwent an EEG test, a warm-up, another EEG test during an 8 min rest, and then a continuous vertical jump test. The tDCS group had an EEG test, a 10 min warm-up, tDCS stimulation with the Halo Sport 2 headset, an 8 min rest with an EEG test, and then a vertical jump test. The HRT group did an EEG test, a 10 min warm-up, a 2 min rest, five 90% 1RM half squats with 1 min intervals, an 8 min rest with an EEG test, and then a vertical jump test. The tDCS + HRT group had an EEG test, a warm-up, tDCS stimulation with the Halo Sport 2 headset, a 2 min rest, five 90% 1RM half squats with 1 min intervals, an 8 min rest with an EEG test, and then a vertical jump test. Given previous research [30,31] indicating the optimal post-activation effect window is 4–10 min, and considering the time required for EEG testing, we chose to complete the EEG test and the vertical jump test within an 8 min rest period.

Participants were instructed to refrain from strenuous exercise and avoid alcohol or caffeine 24 h before the experiment. Participants were encouraged to maintain a positive mental state and ensure adequate sleep. The intervention and vertical jump tests were conducted in a controlled environment at an independent training facility to minimize external interference.

### 2.4. Intervention

#### 2.4.1. Transcranial Direct Current Stimulation

**Figure 2:**
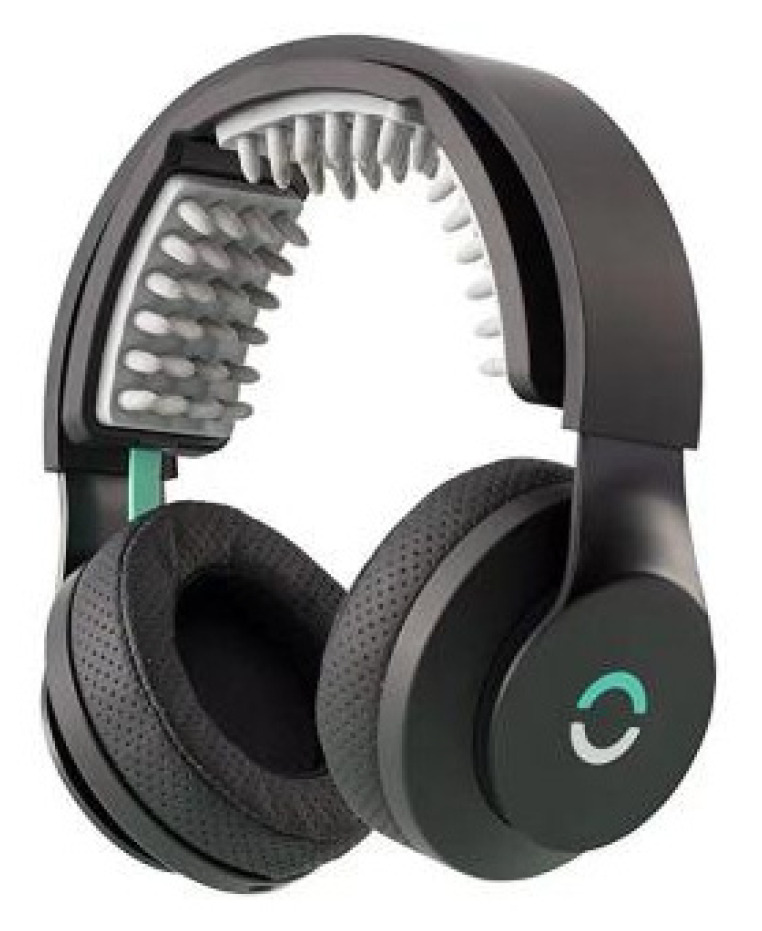
Halo Sport 2 headset.

The Halo Sport 2 headset (HS002K, Halo Neuroscience, Francisco, CA, USA) (Figure 2) delivers a stimulation current of 2.2 mA, with an output frequency ranging from 0 to 625 Hz over a duration of 20 min. The electrodes were positioned over the motor cortex, and the same stimulation parameters were used for all participants to ensure consistency across the study. Previous research [32,33,34] supports the safety and efficacy of this stimulation mode. The electrodes extend across the top of the head from one ear to the other, effectively targeting both hemispheres of the motor cortex. Typically, the anode electrodes are positioned over the top of the motor cortex, while the cathode electrodes are placed at other locations on the head, such as the supraorbital area, to complete the circuit. The current intensity is adjustable via the Halo application, accessible on devices such as iPhones or iPads. During the experiment, participants reclined in a relaxed state in a chair with the Halo Sport 2 headset properly positioned on their heads. Over 30 s, the current was gradually increased to 2.0 mA and maintained for 20 min, following protocols from the existing literature demonstrating improved performance in multiple studies.

Participants had the option to terminate or withdraw from the experiment in case of any discomfort, including head tingling, burning sensations, allergic reactions, significant adverse bodily reactions, nausea, or vomiting.

#### 2.4.2. Lower Extremity High-Load Resistance Training

Following the NSCA testing procedure [29], the participants’ half-squat 1RM was measured with the aim of reaching a 1RM value within five attempts. Post-activation potentiation (PAP) is a phenomenon where the force output of a muscle is temporarily enhanced following high-intensity conditioning activity. Performing sets of five reps at 90% of 1RM can induce PAP, which is believed to enhance neural drive to the muscles, increase motor unit recruitment, and improve muscle fiber synchronization [30,31]. This study utilized 90% of 1RM weight for strength induction, effectively enhancing muscle strength and performance in subsequent explosive activities.

Weight-bearing exercises primarily consisted of half squats and deep squats to induce the HRT effect. The weight-bearing half squat was selected for its capacity to increase gluteus maximus muscle force, alleviate knee joint stress, and mitigate the risk of injury [35]. Thus, the weight-bearing half squat was used to induce the HRT effect in this experiment.

#### 2.4.3. Outcome Measurement Tools

Data collection was conducted by an occupational physiology team with over 3 years of experience, capturing experimental intervention and demographic data for all participants. Importantly, the experimenter remained blinded to the group affiliation of the participants.

To accurately reflect changes in reactive strength without excessively increasing participant fatigue and to ensure data reliability and validity, the Repeat the Vertical Jump 5 times (5-RVT) test was chosen as an indicator of lower limb explosive power [36]. Lower limb explosive strength was evaluated through using the Smart Jump apparatus (9281CA, Kistler Instruments, Hook, UK), known for its high reliability [37]. The test parameters included contact time, flight time (FT), height (H), reaction force index (RSI), and peak power output (PPO). 

Participants were instructed to maintain crossed arms to prevent arm swing interference, focus on a green signal light for initiating the jump, and aim to achieve a fast, high jump with minimal ground contact time. A total of 11 jumps were performed, with only five jumps being counted, excluding the initial pre-jump, and only records with a touchdown time of less than 0.5 s were considered.

#### 2.4.4. Electroencephalography 

**Figure 3:**
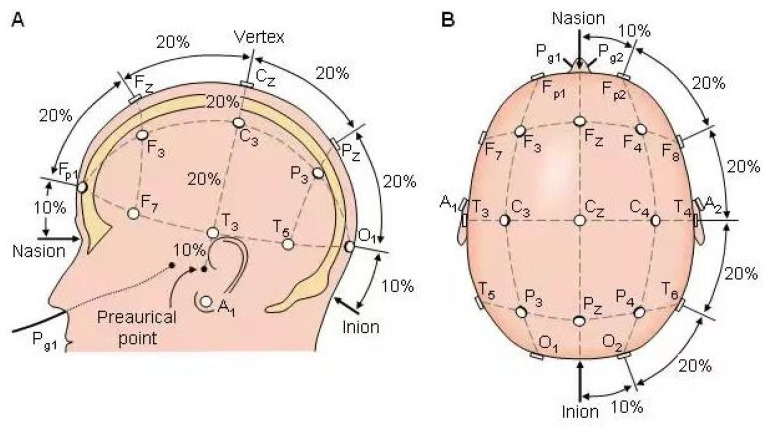
EEG electrode placement diagram. (**A**) Lateral view, (**B**) Top view.

EEG signals were recorded using an EEG test system (Nation9128W, Shanghai Nuocheng Company, Shanghai, China). Electrode placement follows the international standard 10–20 system, positioned on the sagittal plane formed by the connection between the root of the nose and the occipital protuberance [38]. Sixteen electrodes were evenly distributed, with reference electrodes on the bilateral earlobes. The electrodes were placed at Fp1, Fp2, F3, F4, C3, C4, P3, P4, O1, O2, F7, F8, T3, T4, T5, and T6. All electrodes were evenly distributed across all skull positions as shown in the Figure 3A,B. Signals were sampled at 512 Hz, filtered (0.5–30 Hz), and cleaned using independent component analysis (ICA) to remove artifacts. The cleaned signals were segmented into 2 s epochs, and power spectral density (PSD) was calculated using fast fourier transform (FFT). Alpha (8–13 Hz) and beta (13–30 Hz) band powers were averaged for each electrode. Power changes were calculated as percentages relative to pre-intervention values. 

The primary outcome measures included power changes in the alpha and beta frequency bands. Alpha waves (8–13 Hz) are associated with relaxation and reduced cortical activation, while beta waves (13–30 Hz) are linked to active thinking and focus [38]. Changes in these bands provide insights into cortical excitability and neural efficiency following the interventions. Increased alpha power may indicate enhanced neural synchrony and relaxation, whereas increased beta power suggests improved cognitive processing and motor control. By analyzing these changes, we aimed to understand the neural mechanisms underlying the observed improvements in physical performance.

EEG acquisition was conducted in a comfortable, quiet room. Participants were seated in chairs wearing electrode caps and were instructed to remain still.

### 2.5. Statistical Analysis

Statistical analyses were performed using SPSS (version 20.0, IBM Corp, Armonk, NY, USA). Data were presented as mean ± SD. Paired *t*-tests were used to examine potential differences in basic participant characteristics. A two-way ANOVA (Analysis of Variance) was employed to assess the impact of tDCS and high-load resistance training on continuous repeated vertical jump and EEG signals. The “F” in the results refers to the F-statistic from the ANOVA, which indicates whether there are significant differences between groups. Post hoc comparisons were conducted using the least significant difference method, with a significance level set at *p* < 0.05. Additionally, effect sizes were calculated using Cohen’s d (“d”), which measures the standardized difference between two means, providing an indication of the magnitude of the intervention effects.

## 3. Results

Analysis of the baseline data showed no significant differences between groups regarding demographic characteristics (Table 2). Additionally, no adverse events were reported.

### 3.1. Outcome Measures

#### 3.1.1. Repeat Vertical Jumps

##### Contact Time (CT)

A significant interaction effect was observed for the tDCS + HRT intervention (F = 5.33, *p* < 0.05). Significant simple effects were found for both the tDCS (F = 14.90, *p* < 0.001) and HRT (F = 4.90, *p* < 0.05) interventions. Post-intervention, the CT values for the tDCS + HRT condition (0.3 ± 0.24 s) were significantly lower compared to the tDCS (0.4 ± 0.13 s) and HRT (0.4 ± 0.32 s) interventions alone, as well as the CON (0.5 ± 0.03 s) (*p* < 0.05). The Cohen’s d indicated that the tDCS + HRT intervention had a larger effect size (d = −1.17) compared to the tDCS (d = −0.52) and HRT (d = −0.35) interventions. The HRT intervention also had a moderate effect size compared to the CON (d = −0.44).

##### Flight Time (FT)

The tDCS + HRT intervention showed a significant interaction effect (F = 6.33, *p* < 0.05). Significant simple effects were observed for both the tDCS (F = 15.10, *p* < 0.001) and HRT (F = 6.81, *p* < 0.05) interventions. FT values remained consistent across all conditions: CON (0.5 ± 0.04 s), tDCS (0.5 ± 0.02 s), HRT (0.5 ± 0.03 s), and tDCS + HRT (0.5 ± 0.05 s). The SRM for FT showed no significant differences across the interventions (all d ≈ 0).

##### Jump Height (JH)

The analysis revealed a significant interaction effect for the tDCS + HRT intervention (F = 17.12, *p* < 0.001). Significant simple effects were found for both the tDCS (F = 16.85, *p* < 0.001) and HRT (F = 4.78, *p* < 0.05) interventions. Post-intervention, JH values were significantly higher for the tDCS + HRT condition (42.7 ± 3.01 cm) compared to the tDCS (39.6 ± 3.19 cm) and HRT (40.1 ± 3.03) interventions alone, as well as the control group (36.5 ± 3.04 cm) (*p* < 0.05). The SRM indicated a larger effect size for tDCS + HRT (d = 2.05) compared to tDCS (d = 1.0) and HRT (d = 0.86). The HRT intervention also showed a significant effect size compared to the CON (d = 1.18).

##### Reactive Strength Index (RSI)

The tDCS + HRT intervention demonstrated a significant interaction effect (F = 9.33, *p* < 0.001). Significant simple effects were found for both the tDCS (F = 20.30, *p* < 0.001) and HRT (F = 8.33, *p* < 0.01) interventions. RSI values were highest in thetDCS + HRTgroup (2.4 ± 0.03), followed by HRT (2.3 ± 0.05), tDCS (2.2 ± 0.07), and control (2.0 ± 0.07). The SRM showed a significantly larger effect for tDCS + HRT (d = 7.41) compared to tDCS (d = 2.86) and HRT (d = 4.21).

##### Peak Power Output (PPO)

A significant interaction effect was identified for the tDCS + HRT intervention (F = 13.01, *p* < 0.001). Significant simple effects were observed for both the tDCS (F = 10.84, *p* < 0.001) and HRT (F = 4.66, *p* < 0.05) interventions. PPO values were higher in the tDCS + HRT group (45.8 ± 3.53 w/kg) compared to the tDCS (45.0 ± 3.36 w/kg), HRT (41.6 ± 3.57 w/kg), and control (41.5 ± 4.01 w/kg) groups. The SRM indicated a larger effect for tDCS + HRT (d = 1.14) compared to tDCS (d = 0.91) and HRT (d = 0.05).

#### 3.1.2. Electroencephalogram Characteristics Percentage of α-Wave EEG Power

**Figure 4:**
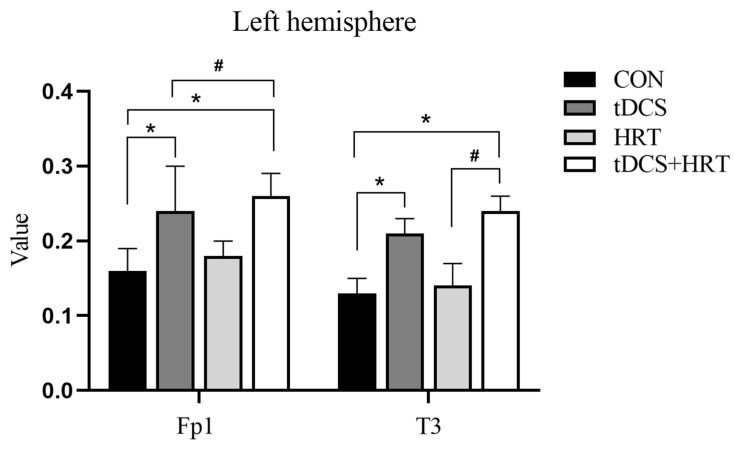
α-wave EEG power. *: Indicates a significant difference compared to CON. #: Indicates a significant difference compared to tDCS + HRT.

An interaction effect of the tDCS + HRT intervention was found to significantly impact the percentage of α-wave EEG power in the frontal lobe area (Fp1) of the left hemisphere (F = 6.33, *p* < 0.05). The Cohen’s d indicated that the tDCS +HRT intervention had a larger effect size (d = 3.33) compared to the tDCS (d = 1.70) and HRT (d = 0.78) interventions. Additionally, a significant simple effect of the tDCS intervention was observed (F = 14.90, *p* < 0.001), while the HRT intervention did not show a significant simple effect (F = 0.231, *p* > 0.05). Similar results were seen in the temporal lobe area (T3) of the left hemisphere, with a significant interaction effect of the tDCS + HRT intervention (F = 6.33, *p* < 0.05). The SRM for the tDCS + HRT intervention was 5.50, indicating a much larger effect size compared to the tDCS (d = 4.00) and HRT (d = 0.39) interventions. A significant simple effect of the tDCS intervention (F = 14.71, *p* < 0.001) was observed, and a nonsignificant simple effect of the HRT intervention (F = 0.24, *p* > 0.05). More detailed information is shown in Figure 4.

#### 3.1.3. Percentage of β-Wave EEG Power

The study found a non-significant interaction effect of the tDCS + HRT intervention on the percentage of β-wave EEG power in the frontal region (Fp1) of the left hemisphere (F = 0.35, *p* > 0.05). However, a significant main effect of the tDCS intervention was observed (F = 17.65, *p* < 0.01), with the Cohen’s d indicating a larger effect size for tDCS (d = 9.74) compared to the control group (CON).

Additionally, a significant interaction effect of the tDCS + HRT intervention was found on the percentage of β-wave EEG power in the frontal region (F3) of the left hemisphere (F = 7.53, *p* < 0.05), along with significant simple effects for both the tDCS (F = 11.10, *p* < 0.001) and HRT (F = 5.23, *p* < 0.05) interventions. The SRM showed that the tDCS + HRT intervention had a larger effect size (d = 8.72) compared to both the tDCS (d = 9.51) and HRT (d = 7.04) interventions.

**Figure 5:**
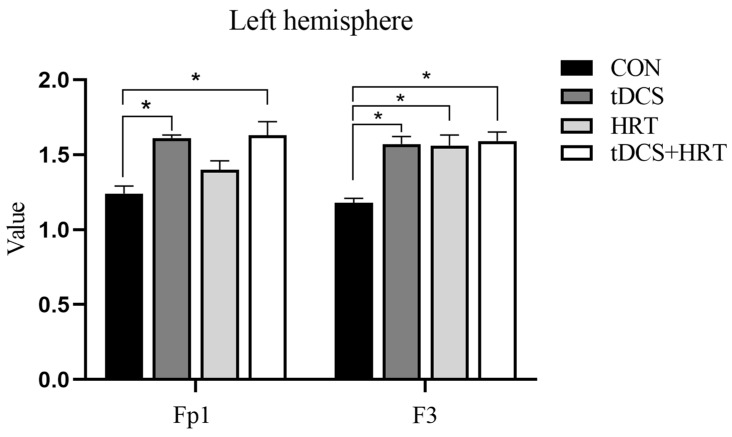
Left hemisphere β-wave EEG power. *: Indicates a significant difference compared to CON.

**Figure 6:**
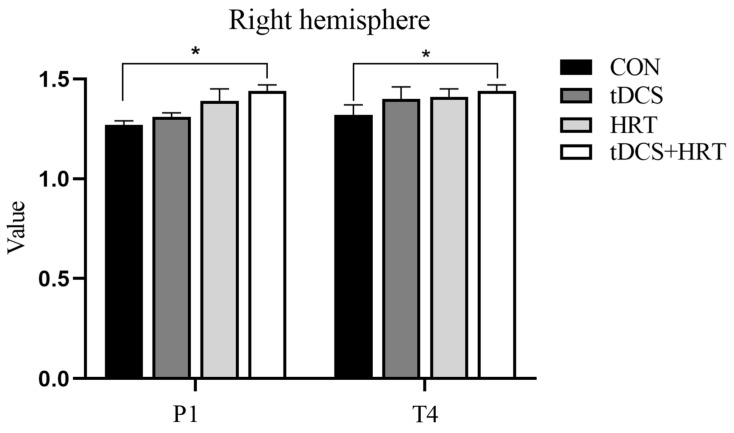
Right hemisphere β-wave EEG power. *: Indicates a significant difference compared to CON.

Moreover, the tDCS + HRT intervention resulted in significantly higher levels compared to the tDCS and HRT interventions (*p* < 0.05), while the tDCS intervention showed significantly higher levels than the CON (*p* < 0.05). Furthermore, the percentage of β-wave EEG power in the right hemisphere parietal region (P4) and temporal region (T4) was significantly higher with the tDCS + HRT intervention compared to CON (*p* < 0.05). More detailed information is shown in Figure 5 and Figure 6.

## 4. Discussion

### 4.1. Effects of Different Interventions on Repetition Vertical Jump 

This study innovatively employed the portable and easy-to-wear Halo Sport 2 headset, incorporating settings commonly associated with positive results in prior research (stimulation current of 2.2 mA, output frequency ranging from 0 to 625 Hz, and duration of 20 min). Unlike most previous studies that focused solely on the effects of single-session tDCS on athletic performance, our research combined tDCS with resistance training and conducted comparative analyses to explore their combined effects. Notably, our study’s innovation lies in integrating tDCS with the post-activation potentiation effect generated by high-load resistance training and investigating its physiological mechanisms through electroencephalography (EEG) changes. This approach not only offers a new perspective on the combination of tDCS and athletic performance but also unveils potential physiological mechanisms, providing a unique and forward-looking contribution to the field.

In this crossover study, we individually investigated the effects of tDCS, HRT, and tDCS + HRT on the 5-RVT and compared these effects within the same participants under control conditions. The results of the experiment demonstrated a significant interaction between HRT and tDCS interventions across various indices such as contact time (CT), flight time (FT), height (H), reactive strength index (RSI), and peak power output (PPO) during the repeated vertical jump. Additionally, both tDCS and HRT showed significant simple effects. These findings suggest that the combination of HRT and tDCS leads to a significant improvement in 5-RVT performance. Consistent with our study findings, Lattari’s research involved 10 participants in a randomized, double-blind study under three conditions [39]. Although Lattari et al. [39] conducted countermovement jumps rather than repeated vertical jumps, their analysis indicated significant increases in jump height, flight time, and peak muscular power following stimulation in the anodal condition. Similarly, Grosprêtre et al. [40] showed that an extracephalic anodal montage (anodal at M1, cathodal on the contralateral shoulder) significantly enhanced jump performance, accompanied by increased excitability at both supraspinal and spinal levels. 

However, some studies, such as those by Romero-Arenas et al. [41] and Park et al. [42] did not observe any improvements in jump performance due to tDCS, possibly due to factors such as the duration of effects, equipment functionality, stimulation sites, and the training levels of the participants. For instance, Marinus et al. [43] conducted a systematic review and found that the influence of a single tDCS session on physical fitness varies significantly based on these factors. Similarly, Chinzara and Harris [20], in their meta-analysis, highlighted the mixed results of tDCS on physical endurance, muscular strength, and visuomotor skills, emphasizing the need for standardized protocols. Hu et al. [44] also noted, in their systematic review and meta-analysis, the varying effects of tDCS on upper limb muscle strength and endurance, suggesting that differences in protocols could explain these inconsistencies. Furthermore, Savoury et al. [45] discussed methodological issues in enhancing muscle strength and endurance with tDCS, underscoring the importance of consistent and well-controlled experimental designs.

Our study builds on these previous works by incorporating a combined intervention of tDCS and HRT, which has not been extensively explored before. We improved upon existing methodologies by carefully controlling variables such as electrode placement, stimulation intensity, and session duration, as recommended by prior studies. This approach allowed us to better isolate the effects of the combined interventions and understand their synergistic impact on lower limb explosive power.

Future research should investigate the long-term impact of tDCS on jumping performance and offer a more comprehensive analysis of lower limb dynamics in vertical jumping. Additionally, exploring the effects of varying tDCS parameters and their interactions with different training protocols could provide deeper insights into optimizing athletic performance enhancements.

### 4.2. Physiological Rationale behind Improved Performance

The rationale behind these results can be suggested from a physiological perspective. The data presented in Table 2 revealed a significant increase in vertical jump height following HRT, which may suggest the recruitment of more muscle fibers and enhanced activation of fast-twitch muscle fibers. It was also possible that the activation of muscle contraction was accelerated, leading to increased muscle output after engaging in half-squat exercises with heavy loads. However, these hypotheses require further investigation as we did not directly measure excitability, nerve impulse frequency, or muscle fiber recruitment. Furthermore, the results from Table 2 indicated that RSI and PPO were maximized post-tDCS intervention. These parameters were closely linked to the functioning of the nervous system, suggesting that tDCS might enhance neuromuscular efficiency. Again, further studies are needed to confirm these findings and to explore the underlying mechanisms more thoroughly. Previous research has highlighted that tDCS could expedite the regulation of spinal cord nerves, elevate the speed of muscle contraction, and enhance the coordinated control of lower limb muscles [46,47]. Stimulation of the motor cortex through tDCS augments motor neuron activity and the excitation of tendon organs, resulting in enhanced afferent impulses during muscle contraction. Overall, the enhancements observed in all 5-RVT indices following tDCS combined with HRT intervention signify increased nervous system excitation, frequency and intensity of nerve impulses, enhanced muscle fiber recruitment, heightened activation, and accelerated contraction rate, ultimately leading to an improved 5-RVT performance.

Although the tDCS Halo Sport 2 headset, developed based on the principles, has been shown to positively influence sports performance in sports science studies, there are scholarly inconsistencies. For example, Fortes et al. suggested that tDCS does not improve power and that its biggest influence is the individual’s training experience and level [48]. Individuals with no training experience had a more significant effect after being stimulated with tDCS than those with training experience, possibly due to the fact that neuromuscular function is more optimized (e.g., peak power and volume of training) in participants with training experience [49]. Lerner et al. [50] have noted that tDCS produces ‘noise’ in the motor cortex of the brain when stimulated, thereby attenuating motor performance. One study found that anodal tDCS stimulation of the M1 region decreased participants’ pain perception after stimulation of the M1 region, as observed by international 10–20-lead EEG, and helped athletes to reduce fatigue during both endurance and high-load resistance training [51].

The level of activation in the motor cortex may have reflected reflect the underlying mechanisms of enhanced motor performance. Factors influencing brain wave frequency include the duration of neuronal circuit activity, the number of synapses transmitting nerve signals, and the metabolic rate of neuronal substances [52]. An increase in the alpha frequency band suggests that the firing rate of the central nervous system is becoming more coordinated and synchronized, indicating improved stability in EEG changes [53,54]. The study results demonstrated a significant rise in alpha band power following both tDCS + HRT and tDCS interventions, suggesting that tDCS interventions could enhance the organization of the central nervous system in terms of synchronization, desynchronization, and modulation between inhibition and excitation. Furthermore, the 5-RVT results revealed significant increases in CT, RSI, and PPO indices after tDCS and tDCS + HRT interventions, all of which were strongly correlated with nervous system function. This could explain how tDCS led to improved 5-RVT performance.

β-waves played a crucial role in indicating neural activity. β-waves are linked to the level of nerve cell activation in the cerebral cortex [55]. Proper activation suggested the brain was appropriately aroused, while excessive activation may indicate tension [56]. The study’s findings revealed that the percentage of power in the β-frequency band after the HRT and tDCS + HRT interventions was significantly higher than that of CON (*p* < 0.05) for each, and the β-frequency band after the tDCS + HRT intervention was higher than that of tDCS (*p* < 0.05). These changes could be attributed to both intervention protocols involving a demanding load resistance exercise (90% 1RM half squat). Research has highlighted that β-wave alterations are particularly responsive to exercise intensity [57].

### 4.3. Cognitive Functions and Brain Synergy

The prefrontal lobe, which makes up 25% of the entire cerebral hemisphere area, plays a crucial role in higher cognitive functions such as attention, memory, problem solving, and personality development [58,59,60,61,62]. Engaging in cognitive activities has been shown to enhance brain organization [63]. Results from this study indicated that the primary brain areas with the highest EEG power percentage in the α-band were the frontal area (Fp1) and lobe area (T3) in the left cerebral hemisphere. Changes in the β-waves were observed in the central area of the left cerebral hemisphere (C3) and the frontal area (Fp2). Despite variations in left–right brain synergy following stimulation, with a tendency towards dominance in the left side, the frontal regions of both hemispheres exhibited more stable synergy under specific conditions. Brain synergy is a critical mechanism for assessing brain activity, and all human cognitive processes are essentially organized brain functions under the influence of synergy [64]. The Halo Sport 2 delivers stimuli to the M1 area of the primary motor cortex via electrodes integrated into the headset, targeting the primary active brain area identified in this study. The M1 area is responsible for generating nerve impulses that travel down the spinal cord to control human movement execution and enhance cortical excitation [38]. Therefore, the enhanced repeated vertical jump performance observed after tDCS and tDCS + HRT may be linked to changes in EEG frequency bands. However, improvements in motor performance are multifaceted and can also be influenced by individual participants’ central cerebral state, emotions, training level, and other factors [65]. The results of this study indicate that combining HRT with tDCS intervention can improve central nervous system function.

## 5. Conclusions

The study found that tDCS, HRT, and the combination of both significantly improved continuous repeated vertical jump performance. Notably, the combined intervention of tDCS and HRT was found to be the most effective. Specifically, this combined intervention outperformed transcranial direct current stimulation or resistance exercise alone in enhancing repeated vertical jump performance. The positive effects observed were correlated with the impact of transcranial direct current stimulation on brain electrical activity in various regions, resulting in enhanced alpha and beta waves.

### 5.1. Limitations and Future Directions

In our study, although we found that the combination of tDCS and resistance training significantly enhanced lower limb explosive power, these results may be influenced by the lack of blinding. Therefore, future studies should adopt a double-blind design to enhance the validity of the findings. This can be achieved by using a sham stimulation, where the tDCS device is applied but no current is delivered, ensuring that participants cannot distinguish between the active and sham conditions. Additionally, assessing the success of the blinding process by asking participants whether they believed they received the active or sham treatment can provide further insights into the potential influence of placebo effects.

### 5.2. Future Applications

The potential of combining transcranial direct current stimulation (tDCS) with high-load resistance training (HRT) for sports training is significant. Future research should focus on how to effectively integrate this combined approach into the training cycles of high-level athletes to optimize their performance. Additionally, it is important to explore the impact of this training method on athlete fatigue. By personalizing these interventions and considering factors such as age, training phase, and specific athletic needs, training outcomes can be further enhanced, providing athletes with scientifically validated and effective training programs.

## Figures and Tables

**Table 1:** Basic information on participants.

Number of Participants (n)	Age (yr)	Height (cm)	Body Weight (kg)	1RM Back squat/kg
29	21.8 ± 2.13	178.1 ± 6.15	77.1 ± 7.70	2.15 ± 0.20

**Table 2:** Effect of different intervention methods on repeated vertical jump.

	CON	tDCS	HRT	tDCS + HRT
CT (s)	0.5 ± 0.03 ^▲^	0.4 ± 0.13 *^▲^	0.4 ± 0.32 ^▲^	0.3 ± 0.24 *
FT (s)	0.5 ± 0.04	0.5 ± 0.02	0.5 ± 0.03	0.5 ± 0.05
JH (cm)	36.5 ± 3.04 ^▲^	39.6 ± 3.19 *^▲^	40.1 ± 3.03 *^▲^	42.7 ± 3.01 *
RSI	2.0 ± 0.07	2.2 ± 0.07	2.3 ± 0.05	2.4 ± 0.03
PPO (w/kg)	41.5 ± 4.01	45.0 ± 3.36 *	41.6 ± 3.57	45.8 ± 3.53 *

*: significant difference compared to CON (*p* < 0.05). ^▲^: significant difference compared to tDCS + HRT (*p* < 0.05). Contact time: CT; flight time: FT; jump height: JH; reaction force index: RSI; peak power output: PPO.

## Data Availability

Datasets are available through the corresponding author upon reasonable request.

## Abbreviations

1RM	One Repetition Maximum
5-RVT	Repeat the Vertical Jump 5 Times
C3,4	Central Region 3,4
CON	Control Condition
CT	Contact Time
EEG	Electroencephalographic
F7,8	Frontal Electrode 7,8
Fp1,2	Frontal Lobe 1,2
FT	Flight Time
H	Height
NSCA	National Strength and Conditioning Association
O1,2	Occipital Lobe Region 1,2
P3,4	Parietal Region 3,4
PPO	Peak Power Output
RSI	Reaction Force Index
HRT	High-load Resistance Training
T4,7	Temporal Lobe Region 4,7
tDCS	Transcranial Direct Current Stimulation
